# The Role of Cellular Metabolism in Maintaining the Function of the Dentine-Pulp Complex: A Narrative Review

**DOI:** 10.3390/metabo13040520

**Published:** 2023-04-05

**Authors:** Kacper Nijakowski, Martyna Ortarzewska, Jakub Jankowski, Anna Lehmann, Anna Surdacka

**Affiliations:** 1 Department of Conservative Dentistry and Endodontics, Poznan University of Medical Sciences, 60-812 Poznan, Poland; 2 Student’s Scientific Group in the Department of Conservative Dentistry and Endodontics, Poznan University of Medical Sciences, 60-812 Poznan, Poland

**Keywords:** metabolism, cellular signalling, dental pulp, odontoblast, pulp stem cell, pulp inflammation, diabetes, ageing, dental procedure, anti-inflammatory mediator

## Abstract

The cellular metabolic processes ensure the physiological integrity of the dentine-pulp complex. Odontoblasts and odontoblast-like cells are responsible for the defence mechanisms in the form of tertiary dentine formation. In turn, the main defence reaction of the pulp is the development of inflammation, during which the metabolic and signalling pathways of the cells are significantly altered. The selected dental procedures, such as orthodontic treatment, resin infiltration, resin restorations or dental bleaching, can impact the cellular metabolism in the dental pulp. Among systemic metabolic diseases, diabetes mellitus causes the most consequences for the cellular metabolism of the dentine-pulp complex. Similarly, ageing processes present a proven effect on the metabolic functioning of the odontoblasts and the pulp cells. In the literature, several potential metabolic mediators demonstrating anti-inflammatory properties on inflamed dental pulp are mentioned. Moreover, the pulp stem cells exhibit the regenerative potential essential for maintaining the function of the dentine-pulp complex.

## 1. The Physiological Integrity of the Dentine-Pulp Complex

Extensive, detailed knowledge about the biology, physiology and structure of dentine-pulp complex is necessary in clinical dentistry, which mainly aims to preserve pulp vitality. It also helps clinicians select materials, methods, and techniques in restorative dentistry to provide appropriate treatment. Dental pulp consists of many constituents, such as cells, nerves, blood and lymph vessels, fibres and interstitial fluid. All the components participate in the response to dental procedures. Interstitial fluid, which is mostly similar to plasma, maintains the environment essential to cellular functions [1,2].

### 1.1. Dental Pulp Cells

Many metabolic processes are taking place in the pulp continually. Under physiological conditions, this activity reaches a lower rate; nevertheless, the intensity of metabolic processes increases after irritation with external factors, such as bacteria. One of the first in vivo studies about pulp metabolism was conducted in 1987 by Okiji et al. [3]. Scientists identified the main products of healthy rat dental pulp, such as 6-keto-prostaglandin F1 alpha (6-keto-PGF1α) and 12-hydroxyeicosatetraenoic acid (12-HETE) using high-performance liquid chromatography. A stable metabolite of prostaglandin I2 (PGI2), prostaglandin D2 (PGD2), prostaglandin E2 (PGE2), prostaglandin F2 alpha (PGF2α), and thromboxane B2 (TXB2) were also secreted, but at less than 30% of 6-keto-PGF1α. After that, inflammation was triggered by applying bacterial lipopolysaccharide. The results showed that the production of all these metabolites increased in inflamed rat pulp; however, the highest increase concerned PGE2, which was a 9.3-fold rise in comparison with normal pulp. The authors concluded that arachidonic acid metabolites, including lipoxygenase products, may be involved in the development of inflammation in the dental pulp. This experiment certainly initiated concern about pulpal metabolism issues.

As mentioned earlier, many cellular components, including multipotential mesenchymal stem cells, are found in the pulp, characterised by complicated biological features and promising therapeutic applications. Treatment success depends on the identification of stem cell markers to select the appropriate cell population. Both dental pulp stem cells and stem cells from human exfoliated deciduous teeth share a phenotypic profile of mesenchymal stem cells and express multiple standard markers, including but not limited to CD13, CD29, CD44, CD73, CD90, CD105, CD106, CD146, CD166, CD271, Stro-1, and Stro-3, while negative for CD3, CD8, CD11b (or CD14), CD15, CD19 (or CD79α), CD33, CD34, CD45, CD71, CD117 and HLA-DR. Furthermore, without any stimulation toward differentiation, osteogenic markers, such as osteonectin, osteocalcin, osteopontin, bone morphogenetic protein 2 or 4, runt-related transcription factor 2, and type I collagen, chondrogenic markers, such as type II collagen, adipogenic markers, such as leptin, adipophilin, and peroxisome proliferator-activated receptor gamma, myogenic markers, such as desmin, myogenin, myosin IIa, and alpha-smooth muscle actin are demonstrated. Moreover, octamer-binding transcription factor 4, reduced expression protein 1, Sox2, NANOG, forkhead box D3, and lin-28 homolog A, which are transcription factors responsible for pluripotency in early embryos and embryonic stem cells, were found as well. Notwithstanding, more in vivo studies are necessary because most of these markers are known only from in vitro experiments [4].

Other cells pivotal for pulp biology are fibroblasts. They are underrated, despite playing a critical role in immunoregulatory mechanisms, control of inflammation, and dentine-pulp regeneration [5]. In vitro study conducted by Chmilewsky et al. [6] demonstrated human pulp fibroblasts as the first non-immune cells capable of synthesising all complement proteins involved in initiating dentine-pulp regeneration. Pulp fibroblast metabolism is not as intense without bacterial stimulation. However, the presence of Gram-positive bacteria in carious areas causes complement activation. Moreover, cultured human pulp fibroblasts stimulated with lipoteichoic acid (LTA) express all complement components. This study showed membrane attack complex (C5b-9) formation and C5a active fragment production in the absence of plasma proteins. Furthermore, the experiment demonstrated that the activation of complement proteins produced by fibroblasts and the following release of C5a specifically induced pulp progenitor cell recruitment. To conclude, fibroblast cells participate in tissue regeneration by recruiting pulp progenitors via complement activation, which finds fibroblasts as a potential target in therapeutic strategy connected with dentine-pulp regeneration.

### 1.2. Odontoblasts—Primary and Secondary Dentine Formation

Odontoblasts are part of the dentine-pulp complex, which is considered a single functional unit responsible for dentine production, nourishment, and sensory function [7]. Cellular odontoblast metabolism is essential to the physiological and pathological function of the endodontium [8]. One of the components of the dentine-pulp complex is dentine, which is a product of odontoblast metabolism. This metabolism may be interpreted as releasing several building components required for synthesising various types of dentine and secreted substances that induce an immune response in the pulp (chemokines, cytokines).

Odontoblasts are post-mitotic, mesenchymal cells derived from the neural crest [9]. These cells are situated on the tooth pulp’s periphery [10] and are the first cells commonly affected by bacteria from tooth caries [11]. Because of their position, odontoblasts serve as a conduit for delivering nutrients, oxygen and inflammatory cells to the odontoblasts. They also maintain a tight relationship with the living section of the tooth, notably with nerve endings and blood capillaries [12].

Couve et al. [13] found that the localisation and composition of organelles in odontoblasts depend on the cell lifecycle stage, as evidenced by the pre-odontoblast, secretory, mature, and old odontoblast phenotypes. Odontoblasts have a large, oval, eccentrically located nucleus. Except for lysosomes, autophagic vacuoles, Golgi complex, smooth endoplasmic reticulum (SER) and the rough endoplasmic reticulum (RER) in mature odontoblasts are located in mitochondria. These organelles are distributed around the odontoblast to provide energy for the movement of secretory molecules inside and towards the apical pole of the cell process. Additionally, proteins are created in the RER and delivered to the Golgi complex for packaging and distribution. These compounds may serve as substrates for dentinogenesis [8].

Odontoblasts are composed of a large number of junctional complexes between cells, creating a selective barrier that may regulate the interaction between dentine and pulp and vice versa under normal and pathological conditions [7]. Secretory odontoblasts have intercellular junctions and connect with fibroblasts at the subodontoblast region in the dental pulp. This enables the transfer of ions and molecules and communication between cells, as well as the active transport of secretory molecules to the odontoblast processes and their release during the secretory phase by the odontoblasts [14].

Under physiological conditions, cellular metabolism comprises the formation of primary and secondary dentine. During primary dentinogenesis (prior to tooth eruption), the secretory stage of the odontoblasts produces and regulates the mineralisation of the predentine matrix to form primary dentine. At their apical pole, cells release substances that are part of the extracellular matrix (ECM) [15], such as collagen type I and non-collagenous proteins [12], including SIBLINGs—phosphorylated matrix proteins, non-phosphorylated matrix proteins and proteoglycans, which participate in the mineralisation of the dentine matrix [7,16]. The odontoblasts move pulpally as additional matrix is deposited, leaving behind one or more cytoplasmic processes that are encircled by predentine matrix, which later mineralises to increase the dentine width [17]. The biomarkers for active dentine synthesis, dentine matrix protein-1 (DMP-1) and human dentine sialophosphoprotein (DSPP) show that the odontoblast is at the secretory stage [18].

By delivering calcium ions and inorganic phosphate to the mineralising front, odontoblasts actively contribute to dentine mineralisation. Via voltage-gated Ca^2+^ channels (L-type Ca^2+^ channels) at their basal part and Ca-ATPase and Ca-Na exchangers at their apical pole, respectively, odontoblasts may take in and release calcium ions. In contrast, phosphate transport mechanisms need further research [12].

When teeth erupt and crown development is complete, the odontoblast transitions from the secretory to the mature stage. At this stage, odontoblasts significantly decrease their dentine formation activity in order to produce secondary dentine. Physiological secondary dentine formation will lead to a slow reduction in the pulp chamber size as the matrix is deposited circumpulpally [19]. Secondary dentine is formed at a significantly slower rate of 0.4 μm/day, whereas primary dentine is deposited at 4–20 μm/day [12,20,21]. This secondary dentine is similar to the primary dentine in terms of chemical composition and structural organisation. After primary dentinogenesis, the cell essentially enters a resting state, and the restricted secondary dentine development over many years indicates a basic level of activity of the odontoblast in this resting state [12].

The stage of the lifecycle has a considerable impact on the odontoblast metabolism, which in turn has a significant effect on the functioning of the endodontium since odontoblasts secrete metabolites necessary for the synthesis of primary and secondary dentine.

### 1.3. Odontoblasts and Odontoblast-Like Cells—Tertiary Dentine Formation

**Figure 1:**
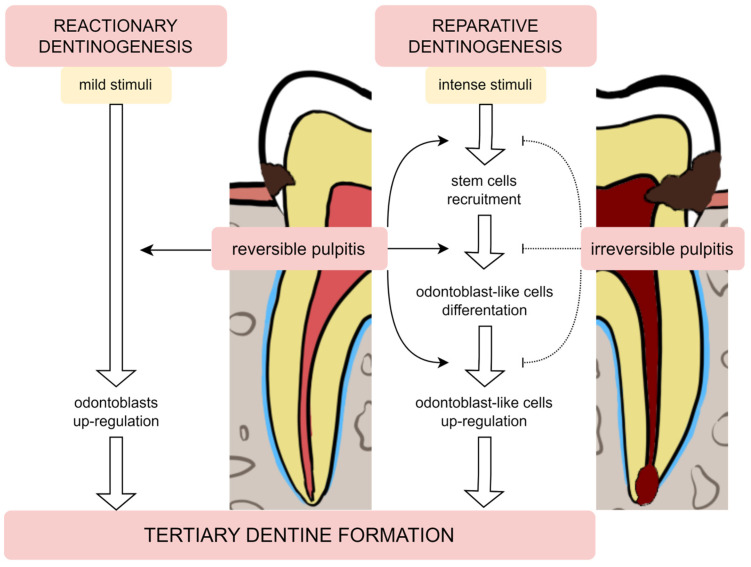
The tertiary dentine formation—reactionary and reparative dentinogenesis during reversible and irreversible pulpitis.

Damage to the tooth tissue leads to the deposition of the tertiary dentine below the injury site [22]. This type of dentine differs from primary and secondary dentine in its composition as well as in the rate of deposition. There are two subtypes of tertiary dentine: reactionary and reparative [21,22]. This classification distinguishes subtypes mainly due to the conditions in which the dentine was formed and the severity of the damaging factor [23]—Figure 1.

Odontoblasts may endure damage produced by mild stimulation, such as slowly increasing caries or attrition, as well as some restorative methods or proinflammatory mediators [10,24]. Odontoblasts create reactionary dentine in response to mild stimulation by upregulating their baseline secretory activity. Moreover, the release of transforming growth factor β1 (TGF-β1) during dentine demineralisation is a significant activator of odontoblast differentiation and dentine matrix secretion [22,25]. This factor is responsible for the increased odontoblast secretory activity [26]. 

In contrast, when the noxious stimulus is stronger (e.g., quickly expanding carious lesion), it can lead to the odontoblasts’ death. To replace these cells, the differentiation of stem cell populations into odontoblast-like cells is initiated. These newly differentiated cells secrete reparative dentine, which has an amorphous structure, an atubular shape and imprisoned cells [26]. The formation of this dentine is a more complicated process than the production of reactive dentine, as it requires the recruitment of pulp cells, their differentiation and induction to secrete the matrix of this dentine [27,28]. 

When the bacteria infect the dentine, it demineralises (as mentioned earlier) and releases dentine matrix components into the dentine-pulp complex [8]. Released growth and angiogenic factors may affect the metabolism of the dentine-pulp complex by promoting angiogenesis or proliferation and differentiation of progenitor cells in the pulp [25,26,29,30]. In addition, some of the substances (DSP) also stimulate the migration and proinflammatory activation of immune system cells [31,32]. However, previous studies mentioned that released dentine matrix components, cytokines and growth factors (TNF-α and TGF-β1) have detrimental effects on pulpal tissue and can cause cellular death [24,32,33,34]. 

It can be assumed that odontoblasts induce pulp cell metabolism indirectly through the secretion of cytokines and chemokines. Nuclear factor kappa B (NF-κB) is an intracellular signalling pathway activated when harmful bacterial substances, such as lipoteichoic acids (LTA), bind to TLR receptors on dental pulp cells like odontoblasts or fibroblasts. The pulp can then generate a cascade of molecules as a result of the binding of cytokines and chemokines to their specific receptors, which will further activate the pulp’s response to infection. The differentiation processes, however, may be inhibited and obstructed due to the activation of the NF-κB proinflammatory signalling cascade [35,36].

The complexity of the reparative dentinogenesis can be a problem in the case of an intense inflammatory response in the endodontium that can hinder the proper course of this process. However, there may be potential beneficial effects of low-grade inflammation on tertiary dentine production, as opposed to the impact of intense and severe inflammation [37,38]. The recruitment of stem cells, development into odontoblast-like cells, and dentine secretion are all supported by the low intensity of the inflammatory mediator of the dentine-pulp complex. On the other hand, some components of the dentine matrix released by bacterial degradation, in addition to pulp cells’ activation of numerous proinflammatory pathways, may initiate intense immune cell activity to clear the infection. Increased immune cell activity may impede the aforementioned healing procedures until inflammation decreases [24].

The influence of the cellular metabolism of odontoblasts on the pulp-dentine complex is particularly visible in pathological conditions. The function of odontoblasts in these conditions is multidimensional and complex.

## 2. The Changes in Metabolic and Signalling Pathways during the Pulp Inflammation

The complications of dental caries are the main cause of triggering inflammatory responses in the dental pulp by the penetration of oral bacteria via the enamel and dentine layers [21]. In response to the release of the metabolically active bacterial components, the dental pulp cells express pattern-recognition receptors, in particular, Toll-like (TLR) and NOD-like receptors (NLR), which can activate nuclear factor-κB (NF-κB) and p38 mitogen-activated protein kinase (MPK) signalling [26].

Also, bacterial invasion is closely related to the enhanced release of a wide range of mediators, especially interleukins and matrix metalloproteinases, from the pulp cells [39]. Surprisingly, the dental pulp may be susceptible to SARS-CoV2 infection, and then the patients present worse outcomes of pulp inflammation [40]. In general, pulp inflammation is strictly associated with lipopolysaccharide (LPS) induction. The bacterial LPS mainly affects NF-κB stimulation via TLR receptor activation but also can regulate reactive oxygen species (ROS) production and DNA methylation [41].

Additionally, severe chronic periodontal disease may negatively influence both cementum and dental pulp functions. In the inflamed pulp, the upregulated expression of IL-17 and IL-1β and autophagy markers LC3B and P62 were observed [42]. In addition, reduced oxygen saturation was found in the pulp of teeth with more advanced periodontitis [43]. The dental pulp cells manifest the dynamic response to hypoxia through the modulation of their metabolism by vascular endothelial growth factor (VEGF) expression (regulated by hypoxia-inducible factor-1α) [44]. Similarly, exposure to tumour necrosis factor-α (TNF-α) promotes apoptosis with upregulated VEGF expression and enhanced NF-κB signalling [45].

In a recent study, Yan et al. [46] analysed the cytokine signalling pathways in dental pulp. The activity of TRAIL, NO, IL3, CXCL12 and IL1A was high in the majority of cells in the dental pulp. The dental pulp stem cells demonstrated the elevated activity of NO, TRAIL, CXCL12, BMP4 and BMP6, whereas pulp cells presented the high activity of CXCL12, BMP4, BMP6, BMP2 and IFN1.

Furthermore, Worsley et al. [47] demonstrated that chronic pulpitis promoted the persistent activation of phosphorylated extracellular signal-regulated kinase (pERK) and p38 (pp38) bilaterally in the trigeminal nuclei, and their expression could be further elevated in case of inflammation exacerbation. Yang et al. [48] found that the glutamate signalling mediated by vesicular glutamate transporter-2 could be enhanced in the pulpal axons during the inflammation, leading to hyperalgesia. The pain from the inflamed pulp can refer to other oral sites, e.g., tongue via IL-1RI and TRPV1 signalling in the trigeminal ganglion [49].

Pulp inflammation can be related to the different expressions of various biomarkers in pulp tissue [50]. Table 1 presents the selected mediators that can play a potential role in cell-to-cell signalling and cell-mediated immune response during the inflammatory processes in the dental pulp. Interestingly, adipokines (such as leptin, adiponectin, ghrelin) may demonstrate numerous physiological and pathological functions associated with inflammatory and immune mechanisms, cell proliferation differentiation, dentinogenesis or angiogenesis in the pulp tissues [51].

Moreover, the expressed miRNAs can show both positive and negative effects during pulp inflammation processes. In inflamed dental pulp, miRNAs can be upregulated or downregulated in inflamed pulpal tissues. They can regulate pulp cell differentiation, cellular metabolism and signalling, migration and apoptosis [72]. Also, DNA methylation can modulate the inflammatory response of human dental pulp cells by regulating miRNA expression [73]. Similarly, the different directions of the lncRNAs expression in pulpitis [74].

## 3. The Impact of Selected Dental Procedures on Cellular Metabolism in the Dental Pulp

### 3.1. The Orthodontic Treatment

Orthodontic tooth movements induce vasodilatation and remodelling at the cellular level in the dental pulp [75,76]. Yu et al. [77] suggested that the elevated proinflammatory IL-17A secretion could promote pulp remodelling and alterations in the pulp microenvironment.

The orthodontic intrusion could cause metabolic changes in the dental pulp, reflected by the increased activity of aspartate aminotransferase (AST). Veberiene et al. [78] speculated that the orthodontic force application could promote hypoxia following circulatory disturbances in the pulp cells, thereby altering the mitochondrial oxidative phosphorylation system. The study by Perinetti et al. [79] observed that the elevated levels of AST activity in orthodontically treated teeth were comparable with teeth with reversible pulpitis [80].

However, the controlled mechanical forces during orthodontic treatment led to reversible temporary metabolic changes in pulp tissue. These findings were confirmed by the previous studies assessing the AST activity elevations after 7 days of the orthodontic intrusion, which gradually reduced to the control levels [81,82]. Moreover, Chavarria-Bolanos et al. [83] found that the increased expression of substance P and calcitonin gene-related peptide, as well as the increasing expression trends of β-endorphins and methionine-enkephalin, were found in the dental pulp after the controlled orthodontic intrusion, even in asymptomatic teeth without pain.

### 3.2. The Conservative Dentistry Procedures

Via ROS signalling, the BisGMA monomers stimulate prostaglandin E2 (PE2) production by the pulp cells, enhancing their MEK/ERK signalling and leading to the higher production of alkaline phosphatase and IL-8 [84,85,86]. Elevated PGE2 levels accelerate neutrophil infiltration and vascular permeability, causing pulpal inflammation common after the application of composite resin restorations [87,88]. The study by Chang et al. [89] found that the pulpal expression of CES2 could protect against the cytotoxicity of the resin monomers and their metabolites (e.g., methacrylic acid).

Also, the other resin monomers, such as UDMA and TEGDMA, can alter the mitochondrial metabolism of fibroblasts, inducing inflammatory processes in the pulp tissue [90,91,92]. In contrast, the polyacrylic acid released from glass-ionomer cement contains long interconnected and intertwined polymer chains, preventing migration via the dentinal tubules and harmful reactions in the pulp. However, several studies reported the cytotoxicity of HEMA [93,94,95]. Davidovic et al. [96] analysed the impact of various liners on dental pulp in experimental animals. Only in individual cases were the increased vasodilatation and hyperaemia observed, which was explained by the fact of performing the cavity preparation during the restorative procedure.

Based on the systematic review conducted by Rathinam et al. [97], tricalcium silicate cement (e.g., mineral trioxide aggregate or Biodentine) promotes the odontogenic capacity of dental pulp cells via the activation of the extracellular signal-regulated kinase ½, nuclear factor E2 related factor 2, p38, c-Jun N-terminal kinase mitogen-activated protein kinase, p42/p44 mitogen-activated protein kinase, nuclear factor kappa B, and fibroblast growth factor receptor pathways. Silicium ions influence increased metabolism, collagen synthesis, bone mineralisation, and connective tissue cross-linking [98]. Both released calcium and phosphate ions can activate the MAPK signalling pathway, inducing odontoblastic differentiation [99,100].

In a recent study, Mendes Soares et al. [101] evaluated if resin infiltration system components could interfere with pulp metabolism. During infiltrating enamel white spot-like lesions, the hydrochloric acid molecules diffuse via enamel and dentine to the pulp cells, reaching toxic concentrations. It led to a significant reduction in total protein production and alkaline phosphatase activity, as well as decreased cell viability and mineralised nodule formation. These impaired metabolic processes influence on defence abilities of the dentine-pulp complex, involving enhanced mineralisation of the dentine matrix. In response to the etchant application, the mineralisation-related genes for alkaline phosphatase, dentine protein 1 and dentine sialophosphoprotein were downregulated, although they are crucial for the proper defensive dentine formation. Additionally, the significantly upregulated expression of genes for IL-1β and TNF-α was found. Interestingly, the biological effects of the only etchant application demonstrated significantly higher toxicity in comparison with the combined resin application after etching. This may be related to the interaction between the residual resin monomers and unreacted dissociated HCl molecules. In in vitro conditions, the resin infiltration negatively affected the metabolic activity of pulp cells and interfered with dentine-pulp homeostasis. Therefore, although the resin infiltration is considered a minimally invasive conservative dentistry procedure, special care should be taken with lesions reaching the external third of the dentine due to the possibility of diffusion of components into the pulp.

### 3.3. The Dental Bleaching

During bleaching procedures, H_2_O_2_ and its by-products can diffuse to the pulp via the layers of enamel and dentine, triggering the release of inflammatory mediators, increasing vascular permeability, decreasing cellular metabolism, and even leading to pulp necrosis [102,103]. The study by Chen et al. [104] reported that H_2_O_2_ from bleaching gel could induce the pain response via upregulation of transient receptor potential ankyrin 1 (TRPA1) expression in dental pulp stem cells, activating the inflammatory genes TNF-α and IL-6 and the hyperalgesia gene PANX1. The secreted neurotransmitter ATP develops hyperalgesia. In parallel, increased intracellular ROS and calcium ions levels were found.

Da Silva et al. [105] determined that a violet LED affected only the most superficial dental tissues and only accelerated the maturation of dentine collagen fibers without any inflammation and fibrosis processes in the rat pulp tissue after bleaching. Similarly, Barboza et al. [106] observed no impact of the violet LED on dentine collagen biostability. The 17.5% hydrogen peroxidase-based gel used in this study did not influence the pulp collagen fibre maturations compared to the previous studies using 35% concentrations [107,108].

The systematic review conducted by Benetti et al. [109] assessed the bleaching effects on the inflammatory response, as well as the cytotoxicity and cell metabolism of the pulp tissue. The included in vitro studies demonstrated that light could influence pulp cell metabolism. The one using halogen light to activate the bleaching gel indicated negative effects [110], in contrast to three other studies [111,112,113]. During the laser therapy, the light was able to compensate for the cytotoxic effects in one study [114] and was incapable of positively modulating the cell metabolism in two others [115,116]. Low-level laser therapy (LLLT) can stimulate cellular metabolism, collagen synthesis, and angiogenesis [117,118], thereby minimising the oxidative damage of the pulp cells caused by the bleaching agents. According to the beneficial effects of LLLT on pulp cell metabolism, Lima et al. [116] found increased alkaline phosphatase activity when using a lower LLLT intensity (4 J/cm^2^).

Interestingly, Ferreira et al. [119] observed that oxidative stress generates increased levels of IL-6 and TNF-α in the rat pulp tissue, regardless of diabetes mellitus, as well as of IL-17 only in the early periods in normoglycemic ones. After dental bleaching, the normoglycemic group demonstrated an inflammatory response limited to more tissue and cellular disorganisation near the pulp horns.

## 4. The Diabetes-Induced Consequences for the Cellular Metabolism of the Dentine-Pulp Complex

Diabetes, as a systemic metabolic disease, may have an impact on the structure and functionality of dental pulp [120]. In diabetic patients, the dental pulp demonstrates limited collateral circulation and poorer microvasculature with inhibited neutrophil activity, increasing the risk for infection or necrosis via anachoresis [121]. Alsamahi et al. [122] confirmed that the dental pulp in type 2 diabetes presented fibrosis with fewer cells and vessels, thickened vessel walls, more pulp calcifications and collagen depositions. Moreover, the significantly upregulated expression of inflammatory cytokines (IL-1β, IL-6, IL-17 and TNF-α), macrophage and dendritic cell markers (CD68 and CD83) and innate inflammation markers (such as TLR2 and TLR4) was found. In contrast, the expression of FOXP3 (regulatory T-cell marker) was downregulated.

In the experimental model performed by Catanzaro et al. [123], diabetes progression was associated with metabolic alterations in dental pulp tissue. During the initial phase of diabetes, the levels of nitrite, kallikrein, alkaline phosphatase and myeloperoxidase showed significant increases. These changes confirmed that neutrophils and macrophages infiltrated the inflamed pulp. In contrast, collagen synthesis was inhibited by the elevated levels of advanced glycation end products (AGEs).

Inagaki et al. [124] found that higher glucose concentrations increased alkaline phosphatase and osteopontin production. The duration of hyperglycaemia could induce pathologic calcifications in chamber pulp and the formation of thickened predentine layer in the radicular pulp in diabetic subjects. The osteopontin was strongly stained in these zones, suggesting its involvement in these processes.

Nakajima et al. [125] showed that AGEs, associated with diabetic complications, stimulated mRNA expression of S100A8, S100A9, and IL-1β in diabetic rat pulp tissues. It was suggested that the AGE-induced alterations could be associated with the RAGE–MAPK signalling pathway. Previously, it was reported that elevated AGE levels might promote pulp calcification in diabetic rats [126]. In contrast, high glucose levels inhibited the proliferation of human dental pulp cells and their odontogenic differentiation, as well as stimulated ROS production [127]. Therefore, hyperglycaemia plays a negative role in the healing and regeneration of pulp and periapical tissues in patients with diabetes.

Diabetes mellitus can impair dental pulp metabolism by glucose-induced oxidative stress, leading to pathological conditions with the difficulty of repair in pulp and periapical tissues. Leite et al. [128] evaluated the parameters of altered metabolism and antioxidant system in the dental pulp of diabetic rats. Total protein concentrations and peroxidase activity did not differ significantly compared with healthy ones. Catalase activity was significantly increased, and the sialic acid concentrations were significantly decreased in the diabetic dental pulp cells. Diabetes could result in an impaired antioxidant response by the dental pulp tissue.

Similarly, Bagheri et al. [129] detected significantly elevated expression levels of catalase, superoxide dismutase 1 and glutathione peroxidase 1, as well as enhanced total antioxidant capacity levels due to persistent hyperglycaemia in the dental pulp of diabetic rats. The authors performed the treatment with quercetin (one of the most common polyphenols) which normalised the levels of all determined antioxidants. The proposed mechanism of quercetin action includes reducing the activity of cellular antioxidants via glucose level reducing, Langerhans islets preserving and ROS scavenging.

In the study by Milosavljevic et al. [130], the participants with type 2 diabetes demonstrated decreased melatonin levels and increased inducible nitric oxide synthase (iNOS) levels in human dental pulp tissue compared with non-diabetic participants. The pharmacological concentrations of melatonin did not show cytotoxicity against human dental pulp cells. On the contrary, the melatonin administration normalised superoxide dismutase activity and iNOS levels under hyperglycaemia to be comparable to those under normoglycemia, suggesting its antioxidant properties for the human dental pulp cells in patients with type 2 diabetes mellitus.

On the contrary, Lee et al. [131] investigated that overexpression of cellular myeloblastosis (c-myb) stimulated dentinogenesis, autophagy and cell survival via p-AMPK/AKT signalling pathway, even under glucose oxidative stress. On the contrary, the lack of c-myb inhibited the above processes, so diabetes could irreversibly damage the dental pulp cells.

Interestingly, Yagi Mendoza et al. [132] differentiated the pancreatic islets from dental pulp stem cells using a three-dimensional (3D) system. Generated islets allowed the enhancement of glucose-dependent insulin secretion and pancreatic markers expression, as well as upregulate the PI3K/AKT and WNT pathways. Additionally, Suchanek et al. [133] showed the potential of the pulp stem cells from natal teeth to differentiate into insulin-producing cells. These findings could be crucial for stem cell therapy in patients with type 1 diabetes.

Pulp damage caused by diabetes can result in the apoptosis of the odontoblast-like cell line and other pulp cells [134]. Wu et al. [135] demonstrated that hyperglycaemia-induced glucose oxidative stress exacerbated mPTP opening by inhibiting the Akt-GSK3β pathway, which led to odontoblast-like cell line (mDPC6T) apoptosis. This apoptosis is one of the main manifestations of tissue damage brought on by diabetes pathological conditions.

Furthermore, hyperglycaemia negatively impacts odontoblast differentiation. Yan et al. [134] evaluated the differentiation of dental pulp cells into odontoblastic cells by monitoring alkaline phosphatase (ALP) activity, mineralisation, and the concentrations of proteins associated with mineralisation (osteopontin (OPN), osteocalcin (OCN), osteonectin (ON), dentine sialoprotein (DSP) and dentine matrix protein-1 (DMP-1)). High glucose levels significantly reduced ALP activity and mineralised matrix deposition in differentiated primary human dental pulp cells. As the differentiated dental pulp cells, the expression of the OCN, ON, OPN, DSP, and DMP-1 was also suppressed. These results indicate that high glucose inhibits odontoblastic differentiation and mineralisation. In addition, Gronthos et al. [136] discovered that hyperglycaemia reduced the mRNA expression of ALP, ON, and human dentine sialophosphoprotein (DSPP), which are considered markers for odontoblast and osteoblast development. This proves that increased glycemia causes a decrease in the differentiation of odontoblasts and osteoblasts.

Based on an in vitro study, Horsophonphong et al. [127] found that high glucose levels decreased the proliferation and odontogenic differentiation of human dental pulp cells. Additionally, a high glucose level induced ROS production and reduced glutathione (GSH) level, suggesting an imbalance between free radical synthesis and antioxidant defence that may cause cellular damage. These results indicate that the high glucose environment may impact how tooth pulp heals and regenerates in diabetic patients. Lyu et al. [137] investigated the effects of gestational diabetes mellitus (GDM) on odontoblastic differentiation of dental papilla cells via Toll-like receptor 4 signalling in rat models. According to the findings, GDM considerably impacted the development of dentine and odontoblast differentiation in offspring teeth. GDM influenced the odontoblastic differentiation of dental pulp cells by activating the toll-like receptor 4 (TLR4)/nuclear factor-kappa B (NF-ĸB) signalling pathway and inhibiting SMAD1/5/9 signalling, which is a critical signalling pathway for osteo/odontoblastic differentiation.

Hyperglycaemia occurring in diabetes reduces the ability of odontoblasts to form a dentine bridge (formed by reparative odontoblasts) and increases the infiltration of inflammatory cells [138,139]. In addition, it also causes a reduction in the expression of dentinogenesis molecules and thickened predentine layer, which leads to a reduction in the healing process [124,139].

Moreover, hyperglycaemia negatively affects the synthesis of type 1 collagen by young differentiated odontoblasts and pulp tissue. Välikangas et al. [140] examined the in vitro effects of high glucose and insulin on type I collagen synthesis. High glucose levels, but not insulin, inhibit the production of type I collagen in the most mitotically differentiated young human odontoblasts. This study demonstrated that glucose directly affected odontoblast and pulp tissue cells. When odontoblasts actively produce and secrete collagen during primary dentinogenesis, the authors hypothesise that a high dietary glucose level may severely disrupt odontoblast metabolism.

The impact of diabetes mellitus, as an example of systemic metabolic disease, on the metabolism of odontoblasts and dental pulp cells is still not fully understood and requires further research.

## 5. The Ageing Influence on the Metabolic Functioning of the Pulp Cells and the Odontoblasts

Subsequently, Asghari et al. [141] confirmed that long-time exposure to hyperglycaemia could promote pulp cell cycle arrest and senescence. The beta-galactosidase activity and the expression of cyclin-dependent kinase (CDK) inhibitor p21 were upregulated. The authors suggest that the Wnt signalling pathway and related beta-catenin could be the key regulators to inhibit the senescence progression in the hyperglycaemic condition.

Visfatin signalling is commonly related to proinflammatory and protumorigenic processes [142]. In the study by Ok et al. [143], the visfatin levels in human dental pulp increased with age. Also, the visfatin expression was upregulated during the premature senescence activated by H_2_O_2_ in in vitro conditions. The authors suggest the relationship between this adipokine and the senescence of human dental pulp cells by increased NADPH consumption, induced telomere dysfunction, and upregulated senescence-associated secretory phenotype (SASP) gene expression.

The uremic toxin *p*-Cresol (PC) could induce cellular senescence of dental pulp stem cells, reflecting the ageing changes in dental pulp [144]. This exposure promoted the distraction of the cell cycle (with increased Bax protein and decreased Bcl-2 levels) and the inflammatory processes (with elevated IL-6 expression). In addition, the levels of senescence-related markers, such as β-galactosidase, p21, p53, IL-1β and IL-8, were increased. In contrast, the decreased levels of odontoblast differentiation markers (e.g., dentine sialophosphoprotein, dentine matrix protein 1, osterix, alkaline phosphatase) were observed. This study confirmed the previous findings about the decreased activity of odontogenic extracellular matrix markers in human dental pulp cells [145]. Chronic inflammation and enhanced oxidative stress (especially elevated hydrogen peroxide) are responsible for the expression of ageing-related molecules, leading to the premature senescence of pulp cells [146].

Human dental pulp stem cell-derived small extracellular vesicles could prevent irradiation-induced salivary gland hypofunction, performing the metabolism of reactive oxygen related to the progress of cellular senescence [147]. 

There are many concepts regarding the ageing of pulp cells and cells in general. Donald R. Morse compiled several theories describing the ageing of the dental pulp complex in his study from 1991. These odontoblast theories include:

- Watch-spring theory (the aged pulp’s energy may be used up because its odontoblasts and fibroblasts have fewer mitochondria);
- Falling domino theory (odontoblasts shrink and flatten; aged odontoblastic layers have intracellular and extracellular vacuoles);
- Free radicals (oxygen-generated free radicals from mature dental pulp fibroblasts and odontoblasts may age the pulp);
- Cellular loss (with age, there is a loss of dividing pulp cells, including odontoblasts; the connective tissue replaces cells; dentinogenesis is stopped; however, the periodontal ligament holds the tooth in the alveolar bone after the pulp necrosis) [148,149]. Currently, the last theory seems to be the most likely, along with other mechanisms described in more recent studies summarised below.

In addition to these theories, the author observed changes in odontoblasts under the influence of ageing, such as vacuolisation of the odontoblasts, deposition of the fat droplets (it is also possible as a tissue-processing artefact), shrinkage of remaining odontoblasts, deposition of irregular secondary dentine deposition, and increased dentinal tubules loss [149]. Most of the observations made by the author in 1991 were consistent with more recent studies.

Secretory odontoblasts form primary dentine during tooth formation. Odontoblasts lose their secretory capacity after tooth eruption at the coronal dental pulp and enter a mature stage, when they become shorter, flattened cells with thick deposits accumulated within autophagic vacuoles. As part of the autophagic-lysosomal system, these autophagic vacuoles comprise self-digestive pathways regulated by lysosomes [150]. This cellular metabolic mechanism maintains cellular homeostasis by regulating the turnover of long-lived organelles and proteins [151]. Most long-lived cells have baseline autophagy activity, which is regarded as a maintenance mechanism with an anti-ageing role [150,152]. A wealth of evidence supports a significant function for autophagy in cellular ageing and disorders associated with ageing [153]. Moreover, it was found that, compared to dental pulp cells, elderly odontoblasts exhibit a decrease in autophagy [154].

Autophagic vacuole clusters increase in the supranuclear area of human odontoblasts from young patients and are surrounded by lysosomes [150]. The authors speculated that a decline in autophagic activity is connected with increased lipofuscin granule formation. In adult patient odontoblasts, the autophagic vacuoles are filled with heterogeneous material that is mainly made up of lipid and lipofuscin aggregates that build up in various amounts throughout the cell body. In the study by Couve et al. [13], among odontoblasts taken from different age groups (16, 60 and 75 years old), the accumulation of autophagic vacuoles, along with lipofuscin deposition, increased with age. The accumulating lipofuscin is considered a toxic metabolite and may impair cell function by inhibiting lysosomal degradation capabilities. The progressive lipofuscin deposition in postmitotic cells (such as neurons, cardiomyocytes, and human odontoblasts) might be a characteristic of the ageing process [155]. In permanent postmitotic cells, these deposits serve as a potent age-marker since cells have a limited capacity to remove them [156]. Moreover, changes occur in the content, size, and position of autophagic vacuoles throughout the ageing process of odontoblasts. The number of larger vacuoles increases [11], but the activity of acid phosphatase and LAMP2A expression decreases [150,157]. However, the increase in autophagic vacuoles may also be associated with a reduction in lysosomal function [155].

Other aspects of the impact of odontoblast ageing are reduced mitophagy (mitochondrial autophagy) that eliminates damaged mitochondria [157] and a lower capacity of odontoblasts to react to injury [158]. Indeed, clinical studies have shown that the odontoblasts’ response to dentine damage decreases with age [23,150,158]. Furthermore, the studies on replicative senescence in serially subcultured human dental pulp cells demonstrated a decreased expression of odontogenic markers like DMP-1 and DSPP, indicating that age-related changes decrease the activity of dentine apposition and mineralisation in dental pulp cells [145]. 

The thickness of the odontoblastic layer progressively decreases with age, mainly as a result of a decrease in cell size and cell number [149,157,158]. Couve and Schmachtenberg [150] noticed that the number of coronal odontoblasts decreased with ageing. The peripheral coronal pulp odontoblasts were arranged in 4–6 rows in young individuals (10–15 years old). Odontoblasts are mostly arranged in 4 rows in young adults (aged 25–40), but in adults (aged 50–65), they are reduced in size and number but still form the structure of a pseudostratified palisade.

Moreover, the study by Hossain et al. [159] aimed to investigate the relationships between age among different age groups and the number of odontoblasts, subodontoblasts and fibroblasts in the dental pulp. Two age groups were established for the 81 teeth removed (6–25 years and 26–80 years). The number of cells (odontoblasts, subodontoblasts and fibroblasts) in the younger age group was substantially larger (*p*-value < 0.001) than in the older age group. The authors observed that the number of odontoblasts and subodontoblasts correlated significantly with age. As demonstrated by the decrease in the number of odontoblasts and subodontoblasts with age, the loss of cells implies that the pulp potential for tooth healing would be hampered in older people.

**Figure 2:**
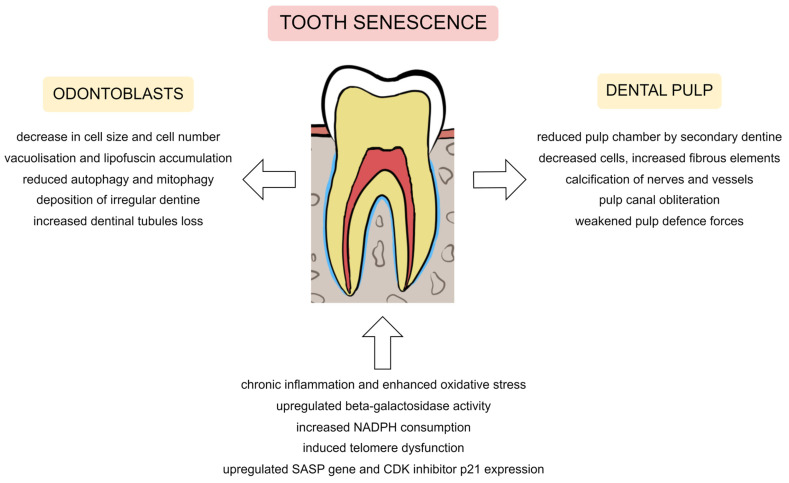
The endodontium senescence manifestations with metabolic and signalling mechanisms. Legend: NADPH, nicotinamide adenine dinucleotide phosphate; SASP, senescence-associated secretory phenotype; CDK, cyclin-dependent kinase.

Therefore, odontoblasts and pulp cells cannot escape the ageing processes. Their content, function, metabolism and quantity change with ageing (Figure 2).

## 6. The Anti-Inflammatory Mechanisms of the Potential Metabolic Mediators on the Dental Pulp

The stemness enhancement of dental pulp cells by short-term Wnt signalling activation (using human recombinant protein WNT-3A) could result in profound metabolic remodelling, especially mitochondrial metabolism with increased tricarboxylic acid cycle activity. It could lead to accumulating reduced power and mitochondrial hyperpolarisation. Also, increased glucose consumption and lipid biosynthesis were determined. The elevated pluripotency core factor expression after Wnt activation caused crucial alterations in both glycolytic and oxidative metabolism [160]. The same activation of Wnt signalling by WNT-3A could induce global DNA hypomethylation and increase histone acetylation and methylation in dental pulp stem cells. These findings indicate that stemness, signalling, metabolic, and epigenetic networks are interrelated in the human dental pulp [161].

In the study by Kornsuthisopon et al. [162], Jagged1-mediated Notch activation upregulated the mRNA levels of the Wnt ligands WNT2B and WNT5A, as well as downregulated the Wnt inhibitors DKK1, DKK2 and SOST. These changes in the mediators of signalling pathways suggest that Jagged1 could promote the odontogenic differentiation of human dental pulp stem cells by modulating both Notch and non-canonical Wnt signalling.

Selected metabolic mediators inhibiting pulp inflammatory processes are presented in Table 2, together with their mechanisms of action. In addition, the interesting metabolite is nitric oxide, which not only regulates the physiological activities of the dental pulp cells but also can initiate and mediate the immune and inflammatory responses triggered by external stimuli [163]. It may play a crucial role in the interactions between inflammatory and regenerative processes in the dental pulp. Moreover, cathelicidins (LL-37) could modulate the pulp innate immune defence system and reparative dentine formation [164].

## 7. The Regenerative Potential of the Pulp Stem Cells for Maintaining the Function of the Dentine-Pulp Complex

There are indications that a new era of restorative dentistry is coming, where regeneration of the dental tissues constitutes a hopeful alternative to traditional restorative techniques. The unconventional approach based on progenitor cells is connected with the promotion of pulp vitality and regenerative therapies. Scientists are aware of the limitations because novel methods are still unpredictable, and the potential of dental pulp progenitor cells is unexplored. Nevertheless, the research is very promising, and the opportunities are constantly increasing [183]. 

In 2000, Gronthos et al. [184] reported for the first time the isolation of a clonogenic, rapidly proliferative population of cells from adult human dental pulp, described as dental pulp stem cells (DPSCs). In the study, DPSCs were compared with known precursors of osteoblasts—human bone marrow stromal cells (BMSCs). DPSCs did not form adipocytes and produced sporadic, densely calcified nodules in vitro; in addition, they generated a dentine-like structure after transplantation into immunocompromised mice. The postnatal dental pulp contains highly proliferative, multipotential cells, which have the ability to form a dentine- or pulp-like structure. Five years later, Shi et al. [185] identified mesenchymal stem cells in human dental tissues, such as adult pulp from third molars, exfoliated deciduous teeth and periodontal ligament. Ex vivo stem cell populations expressed a heterogeneous assortment of markers associated with mesenchymal stem cells, dentine, bone, smooth muscle, neural tissue and endothelium. Researchers found interesting cellular characteristics—similar surface markers (including CD44, CD106, CD146, 3G5, Stro-) and matrix proteins associated with mineral tissue formation (like alkaline phosphatase, osteocalcin, osteopontin), where expressed by dental pulp, bone marrow, and periodontal ligament stem cell populations. However, DPSCs have indicated a higher proliferation rate and growth potential than bone marrow stem cells.

This topic has become very popular in recent years, and many research teams have focused their experiments on stem cells and their potential use in medicine. A series of endogenous and exogenous synergies, encompassing signalling ligands, receptors, pathways and epigenetics, were found to regulate DPSCs metabolism. Development in this branch of knowledge accelerated remarkably when scientists explored the unbelievable potential of pulp regeneration. Available findings and evidence provide an innovative and promising perspective for de novo organ regeneration in the next decades [186]. A huge breakthrough was a study carried out by Iohara et al. [187] in 2004, wherein the team successfully promoted differentiation of DPSCs, receiving pulp-like tissues after pulpectomy. Expression of dentine sialophosphoprotein and enamelysin/matrix metalloproteinase 20 mRNA confirmed the differentiation of pulp cells into odontoblasts and was stimulated by bone morphogenetic protein 2 (BMP2). The authors speculated that BMP2 could direct pulp stem cell differentiation into odontoblasts and result in dentine formation. This team of scientists has spent many years conducting studies related to stem cells. Their previous experiments showed the significant therapeutic potential of DPSCs for pulp regeneration. Due to premises and promising results, Nakashima et al. [188] researched the safety, potential, efficiency, feasibility and predictability of dental pulp stem cell transplantation in teeth after pulpectomy. Studies on five patients with irreversible pulpitis were conducted. DPSCs were transplanted with granulocyte colony-stimulating factor (G-CSF) in atelocollagen into teeth after pulpectomy. Cases were analysed with the electric pulp test, MRI, and CBCT, and the results demonstrated positive pulp response and functional dentine formation. The study proved the safety and efficacy of regenerative methods based on DPSCs with G-CSF; however, trials on a wider group of participants are necessary.

Piva et al. [189] isolated human DPSCs from third molars and expanded them in standard culture conditions containing foetal bovine serum (DPSCs-FBS) or conditions containing human serum (DPSCs-HS) to check optimal, practicable methods for cultivating DPSCs without using xenogeneic components or exogenous growth factors. After 30 days of in vivo experiments on mice, a detailed analysis was used to evaluate blood vessels and dentine formation. Concentrations of angiogenic growth factors produced by DPSCs-HS were comparable to DPSCs-FBS ones. Moreover, several angiogenic factors produced by DPSCs-HS demonstrated higher concentrations in comparison with DPSCs-FBS. The findings showed that DPSCs could maintain pulp regenerative properties without isolation and expansion using animal serum or exogenous growth factors.

Since then, the concept of biology-based treatment has constantly been developing. In 2020, Schmalz et al. [190] tried to analyse and organise the knowledge about options of pulp treatment, especially regenerative approaches in clinical aspects. They prepared a detailed analysis of success rates of classic root canal treatment or pulpotomy (ideas based on the removal of the inflamed or necrotic dental pulp and the replacement by a biomaterial) in comparison with regenerative approaches like revitalisation or regeneration. The authors focused on both mature and immature teeth, likewise different stages of pulp inflammation. The results showed that revitalisation in immature teeth with necrosis is a possible option, as well as an apical plug or coronal regeneration after pulpotomy. Moreover, mature teeth presented a decreased potential for regeneration techniques. Further studies are necessary because regenerative procedures using cell transplantation or cell homing are still in the very experimental phase. Furthermore, rethinking innovative treatment options based on biology and keeping vitality concepts is needed.

The differentiation of dental pulp cells leads to reparative dentine formation, but the biological basis of the mechanism by which dental pulp cells undergo differentiation is still controversial. The signalling pathway during the dental repair process was examined in vivo and in vitro by Li et al. [191]. The goal was to uncover the role of EZH2 (enhancer of zeste homolog 2) and its interaction with the Wnt/β-catenin signalling pathway in the mineralisation of dental pulp cells. The results show that the inhibition of EZH2 promotes the differentiation of human dental pulp cells by activating the transcription of β-catenin, which produces a response in the Wnt signalling pathway, and as a result, mineralisation. This regulation of EZH2 on the Wnt/β-catenin pathway depends on the enrichment of trimethylation on lysine 27 in histone H3 in the promoter region. In addition, Uribe-Etxebarria et al. [160] became interested in canonical Wnt signalling. The scientists examined the metabolic profile of DPSCs during reprogramming linked to Wnt activation by a 48-h exposure to either the GSK3-β inhibitor BIO or human recombinant protein WNT-3A. Significant changes in cell metabolism accompany stem cell differentiation and/or somatic cell reprogramming. Their findings show that increased stemness of DPSCs by Wnt activation comes with a metabolic remodelling, which is clearly described by a crucial participation of mitochondrial metabolism. The authors concluded that stemness and metabolic plasticity are connected because the increase in pluripotency core factor expression observed after Wnt activation in DPSCs was mirrored by valid glycolytic and oxidative metabolism changes.

An essential role in pulp regenerative strategies is played by dental stem cells, such as DPSCs. Both approaches, cell homing and cell transplantation, are dependent on regenerative potential, which is modulated by specific signalling transduction. The reactivation of pivotal signalling pathways in dental stem cells is vitally critical. Liang et al. [192] made an insight from signalling pathways. The authors analysed many studies about pulp regeneration based on cell homing or cell transplantation, in which methods of signalling modulation, including growth factors delivery, genetic modification and physical stimulation, have been used. The findings show that migration, proliferation, odontogenic differentiation, pro-angiogenesis and pro-neurogenesis potentials of dental stem cells can be promoted by cytokines or growth factors, such as stromal cell-derived factor (SDF), fibroblast growth factor (FGF), bone morphogenetic protein (BMP), vascular endothelial growth factor (VEGF) or WNT.

Making tissue engineering strategies more predictable is possible due to maintaining reliable and safe differentiation of stem cells. Huang et al. [193] hypothesised that cell type-specific exosomes could induce lineage-specific differentiation of stem cells. The study evaluated the potential of exosomes from dental pulp cells cultured on undergrowth and odontogenic differentiation conditions to trigger odontogenic differentiation of DPSCs and human bone marrow-derived stromal cells (HMSCs) in vitro and in vivo. The results show that the exosomes can bind to matrix proteins, making it possible to attach to biomaterials. Furthermore, the exosomes induce the enhanced expression of genes necessary for odontogenic differentiation. Thereby, there are prerequisites to using exosomes as biomimetic tools to cause lineage-specific differentiation of stem cells, but further studies are required.

Lambrichts et al. [194] observed that DPSCs are not only able to differentiate into mesodermal lineages (adipo-, chondro-, osteogenic differentiation) but also there is potential for neurovascular characteristics. The team developed a neuronal differentiation protocol and revealed that stem cells could differentiate into both neuronal and Schwann-like cells. Furthermore, stem cells also can constitute an optimal cell source for dental tissue engineering. In addition, the researchers confirmed the formation of both vascularised pulp-like tissue and osteodentine in vivo due to a 3D printing technology. However, further studies are necessary to fully optimise both the innervation and vascularisation potential of DPSCs as well as their hard tissue–forming capacity.

There are many studies comparing dental pulp mesenchymal stem cells with bone marrow ones. Many similarities in morphology, antigens, gene profiles, proliferation and differentiation potentials have been revealed. These two lines of cells were also investigated in vitro and in vivo by Shen et al. [195]. The authors prepared genome-wide RNA sequencing and differential expression analysis, and the results showed differences in adipogenesis, osteogenesis, carcinogenesis, and the PTEN pathway. In this study, dental pulp stem cells presented increased osteogenic and odontogenic potential but decreased adipogenic potential and showed resistance to oncogenic transformation when compared to bone marrow stem cells. Therefore, the enhanced PTEN expression in dental pulp stem cells could be responsible for the lineage commitment and tumorigenesis differences. Also, Yuan et al. [196] investigated the issue of osteogenic differentiation of mesenchymal stem cells, which is essential for bone tissue engineering. They conducted in vitro research to explore the effects of interleukin 10 (IL-10), a well-known anti-inflammatory factor, on the osteogenic differentiation of DPSCs. According to the results, IL-10 could increase the osteogenic differentiation of DPSCs and promote the metabolic switch from glycolysis to oxidative phosphorylation. Researchers concluded that IL-10, a radically important factor for inflammatory repair and bone homeostasis, improves the osteogenesis of DPSCs through the activation of oxidative phosphorylation, which is a potential way to intensify osteogenic differentiation in bone tissue engineering.

Mesenchymal stem cells derived from dental pulp may be a promising model for studying imprinting diseases (IDs), which is a group of rare disorders affecting growth and metabolism. Diseases are mainly because of methylation defects in imprinting control regions that lead to the abnormal expression of imprinted genes. The study by Giabicani et al. [197] aimed to characterise the methylation of imprinting regions in dental pulp stem cells and during their differentiation in osteogenic cells (involved in growth regulation) to assess the interest of these cells in modelling imprinting diseases. The researchers collected dental pulp stem cells from five controls, three patients with Silver-Russell syndrome and one with Beckwith-Wiedemann syndrome. The results showed the imprinting defect in patients and normal profile in controls. Furthermore, the osteogenic differentiation of dental pulp stem cells is an appropriate model for studying imprinting disease pathophysiology. There is a perspective of inventing functional and therapeutic in vitro tests in stem cells from dental pulp and generating other cell types.

**Figure 3:**
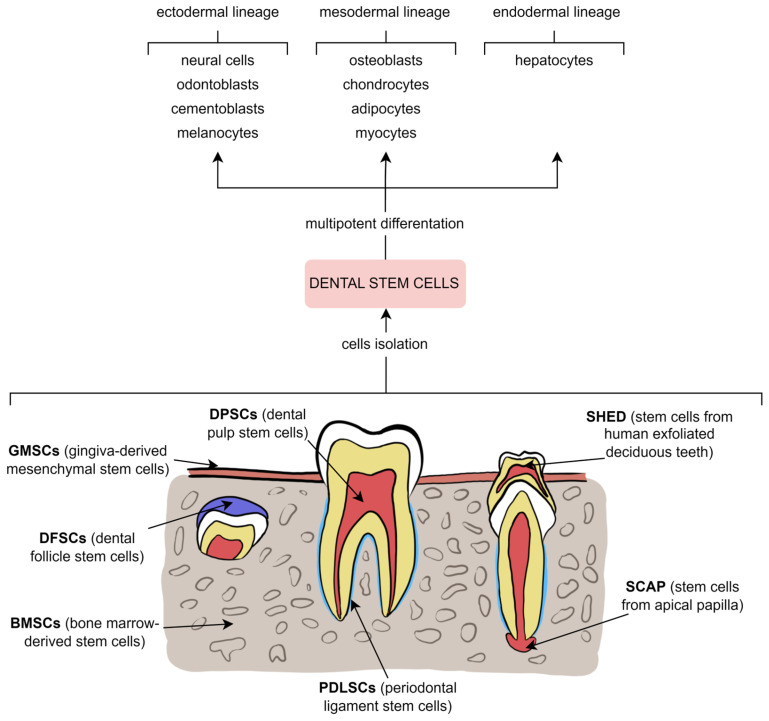
The dental stem cells–the origin and the differentiation.

As mentioned earlier, various types of human dental mesenchymal stem cells are classified by their origin. Moreover, these cells are able to differentiate into many alternative lineages and cells. To clarify, available dental stem cells and possible multipotent differentiation are presented in Figure 3.

Another aspect of keeping pulp vitality, which is crucial in contemporary conservative dentistry, is vital pulp therapy. Direct pulp capping and partial pulpotomy techniques can be used after exposure to vital pulp during caries removal, cavity preparation or accidental injuries. In such situations, hard-tissue barrier formation and, consequently, protection of pulp vitality is expected. Dentine is considered a physiological barrier that can protect the dental pulp from interaction with potential tissue-damaging stimuli. Odontoblasts are the cells responsible for dentinogenesis. Many factors have an influence on therapy success, such as age, health, capping material or type of exposure. Calcium hydroxide (CH), considered for a long time to be the gold standard in these procedures, zinc oxide eugenol, glass ionomer cement, mineral trioxide aggregate (MTA), bioactive calcium silicate-based cement (Biodentine, Septodont), enamel matrix derivative (EMD) are materials used for direct pulp capping. The meta-analysis by Didilescu et al. [198] aimed to objectively evaluate the commercially available pulp-capping materials (MTA and bonding agents) in comparison with CH as a control. The results showed that MTA and CH positively affected hard-tissue barrier formation. However, MTA presented a higher rate of hard-tissue barrier formation than CH. On the other hand, the use of bonding agents was associated with a lower rate of hard-tissue barrier formation than CH. The authors prepare clinical recommendations for using MTA and against using bonding agents in vital pulp therapy.

Vital pulp therapy is a valid trend in conservative dentistry; nevertheless, this method has some limitations, such as healthy pulp necessity. Due to pulp healing limitations, current guidelines and recommendations assume pulpectomy and root canal filling in teeth with irreversible pulpitis. A completely innovative approach was presented by Lin et al. [199]. The authors analysed many studies to provide evidence for the legitimacy of vital pulp therapy of mature permanent teeth with irreversible pulpitis. The scientists suggested a poor correlation between clinical symptoms and pulp sensibility testing and the actual histological status of the pulp. Knowledge about pulp biology, physiology and metabolism is necessary to understand the issue. The injured pulp tissue mobilises undifferentiated mesenchymal stem cells to migrate to the wounded area due to chemokines (e.g., stromal-derived factor-1) releasing. The growth factor is released from the dentine matrix after pulp capping with MTA. Growth factors can signal mesenchymal stem cells to differentiate into odontoblast-like cells and, consequently, produce a dentine bridge. Healing is dependent on infection control, then even irreversible pulpitis in mature teeth appears to be capable of healing. Taha and Abdelkhader [200] conducted an experiment on sixty-four permanent molar teeth with symptomatic vital pulps in 52 patients. After pulpal and periapical detailed diagnosis precise procedure of pulp amputation was done. The researchers used Biodentine (Septodont) as a pulpotomy agent, then resin-modified glass-ionomer liner and ultimately composite or amalgam final restoration. Symptoms indicative of irreversible pulpitis were established in all teeth, and periapical rarefaction was present in nine teeth. After 6 months, 63 of 64 attended recall with 98.4% clinical and radiographic success. Moreover, at 1 year, 59 of 63 attended recall, with 100% clinical and 98.4% radiographic success. In conclusion, pulpotomy with Biodentine is a successful treatment option for mature permanent molar teeth with signs indicative of irreversible pulpitis. In 2017, Asgary et al. [201] presented a multi-centre randomised controlled trial, which aim was to compare success rates of full pulpotomy with two endodontic biomaterials (MTA and calcium-enriched mixture) on symptomatic vital teeth with closed apices. Participants were followed up for 2 and 5 years. The success rates of both groups were above 98%. Regarding radiographic outcomes, after 2 years, MTA was significantly superior to the calcium-enriched mixture; however, after 5 years, success rates were similar. The authors recommend that both biomaterials are effective as capping agents after pulpotomy in mature permanent molars with irreversible pulpitis. This experiment can contribute to a less invasive and more biological approach in dentistry.

To sum up, DPSCs play a valid role in maintaining the function of the dentine-pulp complex. Furthermore, the innovative approach based on stem cells constitutes a promising alternative to traditional restorative dentistry. Many authors indicate possible applications of stem cells in dentistry, such as pulp regeneration in teeth after pulpectomy with irreversible pulpitis or revitalisation in immature teeth with necrosis. Their use in medicine is also wider, for example, in tissue engineering strategies, de novo organ regenerations, as well as a model to study the pathophysiology of imprinting diseases. The research shows that regenerative medicine is relatively new but is developing rapidly, so possible novel applications are expected to be discovered soon.

## 8. Conclusions

Based on our review, cellular metabolism plays a crucial role in maintaining the function of the dentine-pulp complex. Both in inflammatory processes and anti-inflammatory mechanisms, the metabolic mediators perform important functions by regulating the signalling pathways. The factors detrimental to cellular metabolic activity include dental procedures, metabolic diseases (such as diabetes mellitus) or physiological ageing processes. Among the positive aspects, the potential of pulp stem cells in tissue regeneration should be emphasised.

## Figures and Tables

**Table 1:** Selected potential inflammatory and metabolic mediators during the pulp inflammation.

Study	Potential Inflammatory and Metabolic Mediators
Bletsa et al., 2006 [52]	IL-1α, IL-1β and TNF-α (locally produced), IFN-γ and IL-6 (produced systemically)
Brodzikowska et al., 2019 [53]	miR-410 and MMP-14
Feng et al., 2018 [54]	N^6^-methyladenosine, N^6^-adenosine methyltransferase (METTL3) via the NF-κB and MAPK signalling pathways
Gatta et al., 2012 [55]	IL-1β and CD40
Hayama et al., 2016 [56]	kallikrein (KLKB1), protease-activated receptor-1
Huang et al., 2005 [57]	tissue-type plasminogen activator
Hui et al., 2018 [58]	enhancer of zeste homolog 2
Kamio et al., 2008 [59]	plasmin, protease-activated receptor-1
Killough et al., 2009 [60]	substance P
Liao et al., 2019 [61]	sclerostin
Liu et al., 2017 [62]	octamer-binding transcription factor 4-B1
Liu et al., 2021 [63]	lncRNA MEG3
Mehboob et al., 2021 [64]	receptor neurokinin-1
Mente et al., 2016 [65]	matrix metalloproteinase-9
Miyauchi et al., 1996 [66]	PGE2, PGF2, and 6-keto-PGFl
Okiji et al., 1992 [67]	12-hydroxyeicosatetraenoic acid and prostaglandin I2, leukotriene B4
Rethnam et al., 2010 [68]	neuropeptide Y (NPY) Y1 receptor (Y1R)
Sugiuchi et al., 2018 [69]	IL-1β and IL-6, Wnt5a, Runx2, and alkaline phosphatase
Tancharoen et al., 2014 [70]	high-mobility group box 1 (HMGB1), receptor for advanced glycation end products (RAGE)
Wang et al., 2021 [71]	NUTM2A antisense RNA 1, HMGB1

Legend: IL, interleukin; TNF-α, tumour necrosis factor-α; INF, interferon; PG, prostaglandin; NF-κB, nuclear factor-κB; MAPK, mitogen-activated protein kinase.

**Table 2:** Selected potential metabolic mediators with anti-inflammatory interaction against the inflamed pulp cells.

Metabolic Mediators	Anti-Inflammatory Interaction Mechanisms	Study
Nel-like molecule type 1	suppressed expression of proinflammatory cytokines and chemokines (IL-6 and IL-8) mediated via p38 and ERK MAPK signaling pathways	Cao et al., 2021 [165]
Ketoprofen (nonsteroidal anti-inflammatory agent)	suppressed processes, such as IL-1β and TNF-α production, phosphorylation of extracellular signal-regulated kinase and c-Jun N-terminal kinase and the mitogen-activated protein kinase pathway	Choi et al., 2013 [166]
Matrix metalloproteinase 3	decrease in the number of macrophage and antigen-presenting cells, suppressed IL-6 expression; enhanced extracellular matrix formation; modification of serum-derived hyaluronan-associated proteins and hyaluronan (SHAP-HA) complexes possibly via the degradation of versican	Eba et al., 2012 [167]
Taxifolin (natural flavonoid)	increased cell viability and reduced apoptosis; increased carbonic anhydrase IX (CA9) expression	Fu et al., 2021 [168]
Saxagliptin (inhibitor of dipeptidyl peptidase-4)	increased levels of mitochondrial membrane potential (MMP) and adenosine triphosphate (ATP); enhanced processes, such as cell viability and LDH release; suppressed processes, such as ROS production, expression of TNF-α, IL-1β and IL-6, phosphorylation of p38 and activation of NF-κB	Guo and Chen, 2019 [169]
Sirtuin 6 (NAD-dependent protein deacetylase)	suppressed processes, such as expression of proinflammatory cytokines (IL-6, IL-1β and TNF-α) and DMP-1, and activation of NF-κB pathway; enhanced ubiquitination of the TRPV1 channel, leading to its degradation and deactivation	Hu et al., 2020 [170]
Sappanchalcone (flavonoid isolated from *Caesalpinia sappan* L.)	enhanced heme oxygenase (HO)-1 protein expression leading to protect from H_2_O_2_-induced cytotoxicity and ROS production; suppressed release of NO, PGE2, IL-1β, TNF-α, IL-6 and IL-12 in addition to iNOS and COX-2 expression; the transient activation of c-Jun NH2-terminal kinase (JNK) and NF-E2-related factor-2 (Nrf2)	Jeong et al., 2010 [171]
Terrein (fungal metabolite from *Aspergillus terreus*)	suppressed processes, such as ICAM-1 and VCAM-1 expression, AKT phosphorylation and NF-κB translocation	Lee et al., 2008 [172]
Davallialactone (hispidin analogue from the mushroom *Inonotus xeranticus*)	suppressed H_2_O_2_ and ROS production, cellular toxicity and release of inflammatory molecules; restored dentine mineralisation	Lee et al., 2013 [173]
Teneligliptin (inhibitor of dipeptidyl peptidase-4)	enhanced processes, such as overall cell survival and LDH release; suppressed processes, such as ROS production, expression of TNF-α, IL-1β and IL-6, and activation of JNK/AP1/NF-κB signal pathways	Liu et al., 2019 [174]
6-6 bieckol (EB1) and pholorofucofuroeckolA (EB5) from brown seaweed marine algae (*Eisenia bicyclis*)	suppressed processes, such as phosphorylated-extracellular signal-regulated kinase (p-ERK1/2) and phosphorylated-c-jun N-terminal kinases (p-JNK) signalling, NF-κB translocation; enhanced expression of dentinogenic and osteogenic molecules, and dentine mineralisation via ALP activity	Paudel et al., 2014 [175]
Metformin	enhanced processes, such as mineralised nodule formation, alkaline phosphatase activity and expression of odontoblastic markers (DSPP, DMP-1, Runx2 and OCN) via activation of the AMPK signalling pathway	Qin et al., 2018 [176]
Berberine	enhanced cell proliferation; suppressed inflammatory response via miR-21/KBTBD7 axis regulating NF-κB signal pathway	Song et al., 2020 [177]
Phoenixin-20 (via activation of GPR173)	suppressed processes, such as release of proinflammatory mediators (IL-6, MCP-1, VCAM-1, ICAM-1, MMP-2 and MMP-9), activation of TLR-4 and Myd88 and activation of the NF-κB pathway	Sun et al., 2020 [178]
Concentrated growth factor (CGF)	enhanced cell proliferation and mineralisation via activation of the BMP-2/SMAD5/Runx2 signaling pathway; enhanced expression of DSPP, DMP-1, BSP, and ALP	Tian et al., 2019 [179]
Epigallocatechin or epigallocatechin 3-gallate (catechins)	suppressed expression of TNF-α, IL-1β, IL-6 and p-p65 protein, and activation of the NF-κB pathway	Wang et al., 2020 [180]
Let-7c-5p	suppressed DMP-1 expression and NF-κB pathway	Yuan et al., 2018 [181]
Exosomes derived from human umbilical cord mesenchymal stem cells and human dental pulp stem cells	increased proliferation and reduced apoptosis, suppressed release of inflammatory cytokines	Zeng et al., 2022 [182]

Legend: IL, interleukin; TNF-α, tumour necrosis factor-α; LDH, lactate dehydrogenase; ROS, reactive oxygen species; TRPV1, transient receptor potential vanilloid 1; NO, nitric oxide; PG, prostaglandin; NF-κB, nuclear factor-κB; MAPK, mitogen-activated protein kinase; AMPK, AMP-activated protein kinase; iNOS, inducible nitric oxide synthase; COX-2, cyclooxygenase-2; MCP-1, monocyte chemoattractant protein-1; VCAM-1, vascular cell adhesion molecule-1; ICAM-1, intercellular adhesion molecule-1; MMP, matrix metalloproteinase; OCN, osteocalcin; DSPP, dentine sialophosphoprotein; DMP-1, dentine matrix protein-1; BSP, bone sialoprotein; ALP, alkaline phosphatase.

## Data Availability

Not applicable.
